# A comparison of different surgical approaches to hemiarthroplasty for the femoral neck fractures: A meta-analysis

**DOI:** 10.3389/fsurg.2022.1049534

**Published:** 2023-01-06

**Authors:** Liang Shuai, Wu Huiwen, Deng Shihao, Wang Fangyuan, Jing Juehua, Li Jun

**Affiliations:** ^1^ Department of Orthopedics, The Second Hospital of Anhui Medical University, Hefei, China; ^2^ Institute of Orthopedics, Research Center for Translational Medicine, The Second Hospital of Anhui Medical University, Hefei, China

**Keywords:** hemiarthroplasty (HA), anterior approach (AA), lateral approach (LA), posterior approach (PA), dislocation, meta-analysis

## Abstract

There are three traditional surgical approaches to hemiarthroplasty (HA) for femoral neck fractures, respectively, the anterior approach (AA), the lateral approach (LA) and the posterior approach (PA). However, the optimum approach is still controversial, the purpose of this meta-analysis is to identify the merits and demerits of all three approaches. All clinical published studies in PubMed, Web of Science, Embase, and the Cochrane Library from January 2000 to April 2022 were searched which compared different surgical approaches and covered surgery-related outcomes and frequent complications. Five randomized controlled trials and 26 cohort studies for a total of 31 clinical trials were included in the meta-analysis. The dislocation of PA was significantly higher than LA (OR: 3.00 95% CI: 2.25–4.01 *I*^2^ = 27% *P* < 0.00001) and AA (OR: 6.61 95% CI: 2.28–19.13 *I*^2^ = 0% *P* = 0.0005); PA was substantially more than LA in terms of risk of postoperative reoperation (*P* < 0.05); meanwhile, AA has markedly shorter hospital length of stays than LA. The remaining items showed no significant differences in the results.The results of this meta-analysis demonstrated that the risk of PA dislocation and reoperation is higher with hemiarthroplasty, and AA has markedly shorter hospital length of stays than LA.

## Introduction

Femoral neck fractures are a common type of fracture in elderly individuals, accounting for approximately 57%∼64% of hip fractures, with projections estimating the total number of hip fractures worldwide to be approximately 4 million in 2025 (1, 2). Hip arthroplasty is a clinically widespread surgical procedure for the therapy of severely aged femoral neck fractures (3). However, patients with a combination of multiple underlying illnesses, poor health conditions and lower functional demands are more appropriate for artificial femoral head hemiarthroplasty, which can reduce the operation time and allow patients to move early after surgery (4–6).

There are various approaches to hemiarthroplasty, such as the direct anterior approach (DAA) (7), the lateral approach (8), and the true posterior approach (9). Different approaches impact the patient's early postoperative mobility and the occurrence of complications (10). We took van's categorization methodology and classified all the diverse approaches inductively as the anterior approach (AA), the lateral approach (LA) and the posterior approach (PA) (11). Some clinical research has compared two or three of these approaches, but the specific approach with absolute superiority remains indistinguishable, and some authors have tried to perform an analysis using meta-analysis (11, 12), but the number of reports is low and not exhaustive, and the current evidence needs to be updated. Therefore, we performed a meta-analysis comparing the three approaches to examine their possible advantages in terms of complications, postoperative functional outcomes, and surgical outcomes.

## Materials and methods

### Methodology

This systematic review adhered to the preferred reporting items for systematic reviews and meta-analyses (PRISMA) statement. The literature search strictly followed the PICO (Participant, Intervention, Comparison, Outcome) principles. All personnel in this study were involved in designing the search strategy and were trained in search knowledge. A comprehensive search of known databases was conducted to find suitable articles for analysis.

We performed a literature search for studies published in PubMed (*n* = 233), EMBASE (*n* = 474), Web of Science (*n* = 181), and the Cochrane Central Register of Controlled Trials (*n* = 63), finding relevant research published from January 2000 to April 2022, regardless of language. Search for their subject terms and free words of “hemiarthroplasty”, “approach” and “hip”, and various combinations of related words, with the exact search formula referenced to van der Sijp (11). Through the combination of subject words and free words, the articles were screened comprehensively.

### Data abstraction

Two separate writers entered data taken from the included research. Any differences were settled by consensus or dialog with the senior author. Titles and abstracts were reviewed, and inconsistencies were subjected to a full-text search to determine eligibility and settle conflicts by consensus. Sample size, research design, patient age, kind of operation, follow-up data, and outcome data were all retrieved (i.e., surgical outcomes, complications, clinical outcomes).

### Quality assessment

Two writers independently assessed the risk of bias in the papers that were included; if there was a disagreement regarding the results, it was addressed through conversation or with the help of a third investigator. The quality of the methodology of the enrolled research was assessed on the basis of the Cochrane Quality Assessment Form provided by Review Manager (RevMan) 5.4 software in six main areas: selection bias, performance bias, detection bias, attrition bias, reporting bias, and other biases (13). Each type of bias was judged as low risk, high risk, or unclear risk, and a risk of bias map was generated.

### Statistical analysis

Review Manager (RevMan) [Computer program]. Version 5.4.1, The Cochrane Collaboration, 2020, was used to analyze the selected studies. For data extraction, the basic information mainly included study type, follow-up time, sample size of control and intervention groups, age, body mass index (BMI), etc. Specific indicators were surgical outcomes, complications, and clinical outcomes.

The standard deviation (SD) was utilized to compute the mean difference (MD) or Std. mean difference (SMD) and 95 percent confidence interval for continuous data such as length of hospitalization, operation time and surgical blood loss. The odds ratio (OR) and 95% confidence interval (CI) were used for dichotomous data. The *χ*^2^ test result and the value of *I*^2^ were used to examine statistical heterogeneity.

Different authors provide various figures, including medians, ranges or quartiles, and we converted the desired results into means and standard deviations by referring to the statistical method introduced by Wan (14), which is an improvement on the one provided by Hozo and Bland (15, 16), thus bringing the individual values closer to the true value of the data itself and minimizing errors.

To begin, *I*^2^ was used to test the heterogeneity of the results of the included literature; if *I*^2 ^> 50%, *P* < 0.1, which means that there is a large heterogeneity in the included studies; we will explore the reasons for the heterogeneity and conduct sensitivity analysis. In case it is still impossible to eliminate the heterogeneity of the literature, providing clinical consistency, then use the random effect model. If the heterogeneity of the literature was low, a fixed effects model was used.

## Results

**Figure 1:**
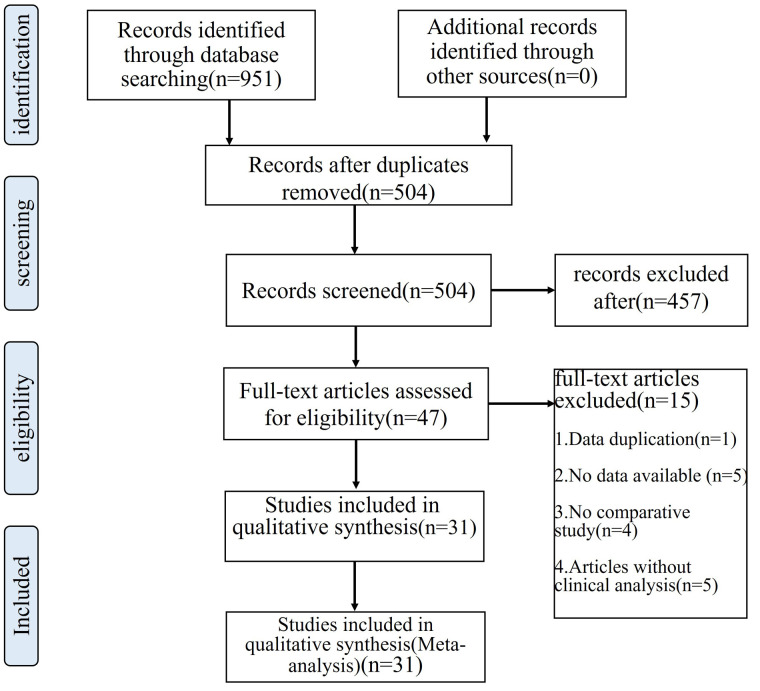
Literature screening flow chart in detail.

A series of 951 relevant articles were accessed in the search database for this research, and after analysis of titles, abstracts, and full text, 31 studies involving the hip were qualified and incorporated into the definitive meta-analysis Figure 1. Detailed descriptions of the characteristics and patient demographics for each study are listed in Table 1. Five were randomized controlled trials (5, 17–20) (RCTs) including 588 hips and the others were cohort studies.

**Table 1:** Characteristics of the included studies.

Author (Year)	Type of study	Approach (Sample Size)			Mean Age (y)	Sex (M/F)	Journal
		AA	LA	PA			
Abram (21) (2015)	RCS	–	753	54	83	232/575	Injury
Auffarth (20) (2011)	RCT	24	24	–	82.6/83.7	10/38	J Trauma
Baba (22) (2013)	PCS	40	–	39	76.7/74.9	15/64	World J Orthop
Bibber (23) (2012)	RCS	–	217	487	80.4/80.3	212/492	Int Orthop
Bűcs (24) (2021)	RCS	51	43	–	79.39/79.26	35/59	Injury
Bush (25) (2007)	RCS	186	–	199	78.34/80.34	102/273	Orthopedics
Carlson (26) (2017)	RCS	85	75	–	82.7/82.9	63/97	Orthopedics
de Vries (27) (2020)	RCS	–	493	516	87/86	NA	Eur J Orthop Surg Traumatol
Enocson (28) (2008)	PCS	–	431	308	84/84	241/929	Acta Orthop
Gursoy (29) (2019)	RCS	–	64	48	87.1/86.5	34/78	Clin Interv Aging
Hongisto (30) (2018)	PCS	–	151	118	82.9/82.5	57/212	Scand J Surg
Kristensen (31) (2017)	RCS	–	18,918	1990	83/83	5,714/15,194	Acta Orthop
Ladurner (10) (2021)	RCS	79	158	–	85.5/86.0	68/196	Arch Orthop Trauma Surg
Lakhani (32) (2022)	RCS	40	54	–	85.34/85.83	30/64	Eur J Orthop Surg Traumatol
Langlois (33) (2015)	PCS	38	–	44	86/85	21/61	Acta Orthop
Leonardsson (34) (2016)	RCS	–	1,140	978	85/85	149/1,569	Bone Joint J
Mansouri (35) (2021)	RCS	–	99	55	77.97/75.43	63/91	Int J Burns Trauma
Mukka (36) (2016)	PCS	–	102	83	83.5/85.51	56/129	Traumatol Surg Res
Neyisci (37) (2020)	RCS	56	–	54	83/82	59/51	Med Sci Monit
Ozan (38) (2016)	RCS	–	86	147	78.3/78.7	97/136	Int J Clin Exp Med
Pala (39) (2016)	RCS	55	–	54	89/87.6	21/88	Eur J Orthop Surg Traumatol
Parker (19) (2015)	RCT	–	108	108	84.3/83.6	20/198	Injury
Renken (18) (2012)	RCT	30	27	–	84/87.5	7/50	BMC Musculoskelet Disord
Saxer (5) (2018)	RCT	99	82	–	84.4/84.0	52/129	BMC Geriatr
Sayed-Noor (40) (2016)	PCS	–	24	24	83.4/82.7	9/39	J Orthop Trauma
Sierra (41) (2006)	RCS	–	1,657	245	63	NA	Clin Orthop Relat Res
Svenøy (42) (2017)	PCS	–	397	186	82.6/83.2	149/434	Injury
Tsailas (43) (2021)	RCS	–	50	50	80.8/82.3	29/71	Injury
Tsukada (44) (2010)	PCS	44	–	39	80.4/81.9	15/68	J Orthop Sci
Verzellotti (17) (2020)	RCT	42	–	44	85.3/85.0	30/70	Hip Int
Yazdanpanah (45) (2020)	PCS	20	–	40	79.27	27/33	Rev Latinoam Hiperte

RCT, randomized controlled trials; PCS, prospective cohort studies; RCS, retrospective cohort studies; NA, not available.

**Figure 2:**
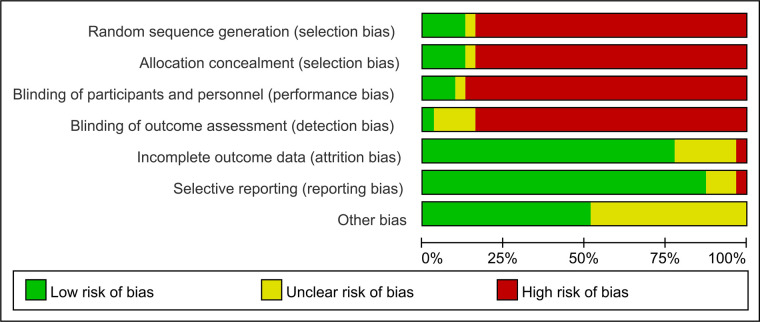
Risk of bias graph.

The danger of selection bias was considerable since just five of the included studies were assigned randomly, accounting for approximately 16% of the total. Because there were so few participants and personnel blinding procedures were used, performance and detection biases were very substantial. The risk of attrition and reporting bias was low, but the risk of other biases was moderate, as shown in Figure 2.

### Length of hospitalization

**Figure 3:**
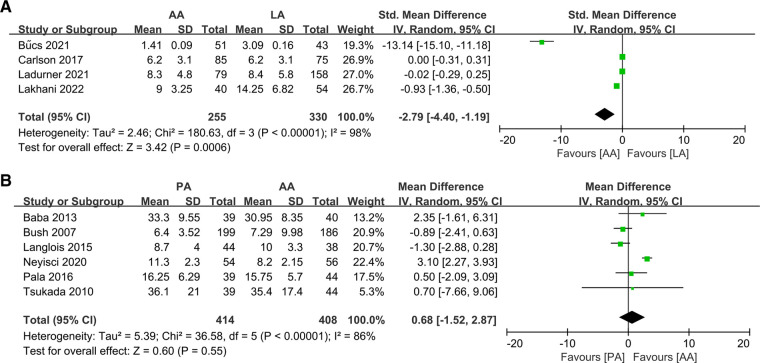
Forest plot comparison of length of hospitalization. (**A**) Anterior approach and lateral approach, (**B**) Anterior approach and posterior approach.

Regarding patient length of stay, four studies (10, 24, 26, 32) reported results comparing AA with LA as well as six studies (22, 25, 33, 37, 39, 44) showed results for PA and AA. The meta-analysis showed a statistically significant difference in length of stay when comparing AA with LA (SMD: −2.79 95% CI: −4.40–1.19 *I*^2 ^= 98% *P *= 0.0006 Figure 3A). There was no significant difference between PA and AA (MD: 0.68 95% CI: −1.52–2.87 *I*^2 ^= 86% *P *= 0.55 Figure 3B). Nevertheless, the number of reported cases for PA and LA was too small, and only de Vries (27) (*P* = 0.58) and Tsailas (43) (*P* = 0.25) were compared, both without differences.

### Operation time

**Figure 4:**
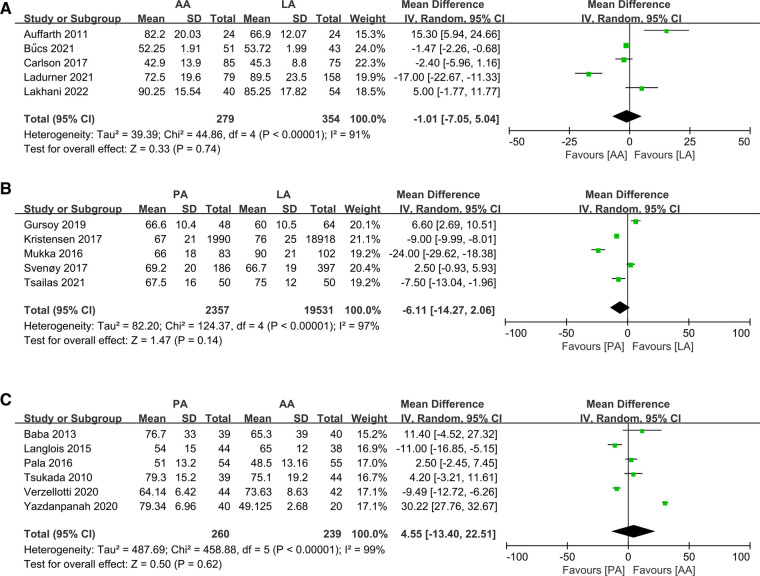
Forest plot comparison of operation time. (**A**) Anterior approach and lateral approach, (**B**) Posterior approach and lateral approach, (**C**) Posterior approach and anterior approach.

Sixteen studies reported the operation time of the 2 methods. The analysis showed no statistically significant differences in all comparisons of operative times: AA vs. LA (10, 20, 24, 26, 32) (MD: −1.01 95% CI: −7.05–5.04 *I*^2^ = 91% *P *= 0.74 Figure 4A); PA vs. LA (29, 31, 36, 42, 43) (MD: −6.11 95% CI: −14.27–2.06 *I*^2^ = 97% *P *= 0.14 Figure 4B); PA vs. AA (17, 22, 33, 39, 44, 45) (MD: 4.55 95% CI: −13.40–22.51 *I*^2^ = 99% *P *= 0.62 Figure 4C). All *I*^2^ ≥ 50% and there was statistical heterogeneity, hence the random effects model was used for analysis.

### Surgical blood loss

**Figure 5:**
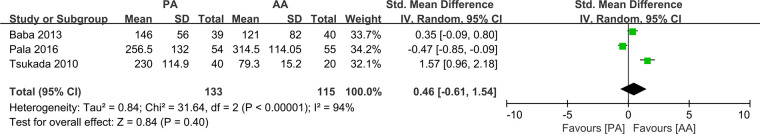
Forest plot comparison of surgical blood loss.

Numerous reports have compared surgical blood loss in different methods, including intraoperative blood loss or transfusion in different units, changes in preoperative and postoperative hemoglobin levels, transfusion rates, and hematoma formation. However, only comparative studies of AA and PA could be converted to uniform units for analysis (22, 39, 44). The outcome demonstrated no significant difference in surgical blood loss between the PA and AA groups (SMD: −0.46 95% CI: −0.61–1.54 *I*^2^ = 94% *P *= 0.40 Figure 5).

### Dislocation

**Figure 6:**
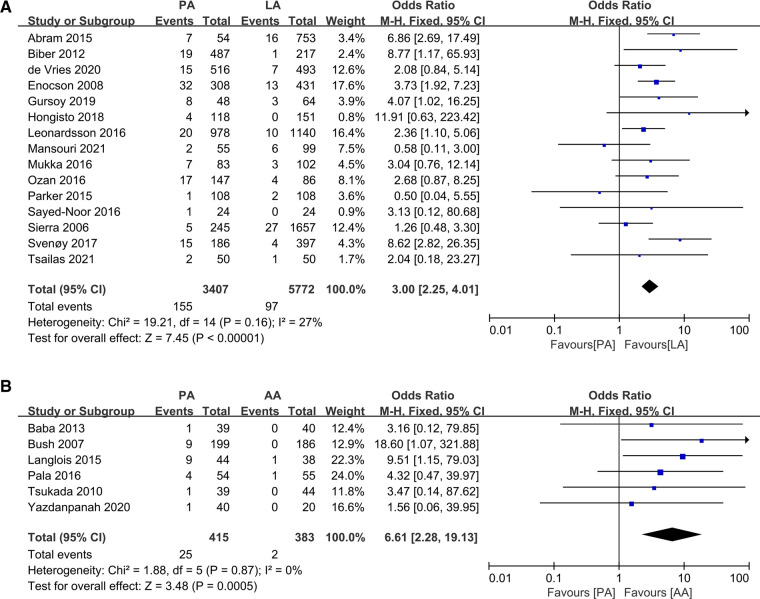
Forest plot comparison of dislocation. (**A**) Anterior approach and lateral approach, (**B**) Posterior approach and anterior approach.

Postoperative dislocation rates were reported in all 15 papers (19, 21, 23, 27–30, 34–36, 38, 40–43), and heterogeneity analysis showed no significant heterogeneity with *I*^2^ = 27% *P = *0. 16, and meta-analysis was conducted using a fixed effects model. The data showed a statistically significant difference in the dislocation rate between the PA and LA groups (OR: 3.00 95% CI: 2.25–4.01 *I*^2^ = 27% *P *< 0.00001 Figure 6A). Simultaneously, after analyzing the data provided by the six articles comparing PA and AA (22, 25, 33, 39, 44, 45), a statistically significant difference was found (OR: 6.61 95% CI: 2.2819.13 *I*^2^ = 0% *P *= 0.0005 Figure 6B). As can be seen, the rate of dislocation was significantly higher in the PA group than in the other two groups. Carlson (26) and Lakhani (32) reported complications of DA and LA dislocation, and although statistical analysis could not be performed, no significant differences were detected on either side.

### Wound infections

**Figure 7:**
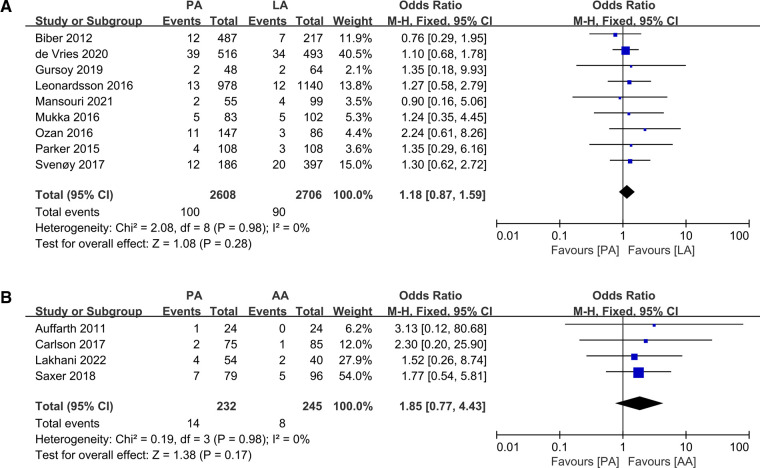
Forest plot comparison of wound infections. (**A**) Posterior approach and lateral approach, (**B**) Posterior approach and anterior approach.

All four studies (5, 20, 26, 32) concluded that no statistically significant difference in the risk of infection was observed between PA and AA, and we did not detect a statistically significant difference when we performed an aggregate analysis (OR: 1.85 95% CI: 0.77–4.43 *I*^2^ = 0% *P *= 0.17 Figure 7A). We obtained the same conclusion in the PA and LA studies (19, 23, 27, 29, 34–36, 38, 42) (OR: 1.18 95% CI: 0.87–1.59 *I*^2^ = 0% *P *= 0.28 Figure 7B).

### Fractures

**Figure 8:**
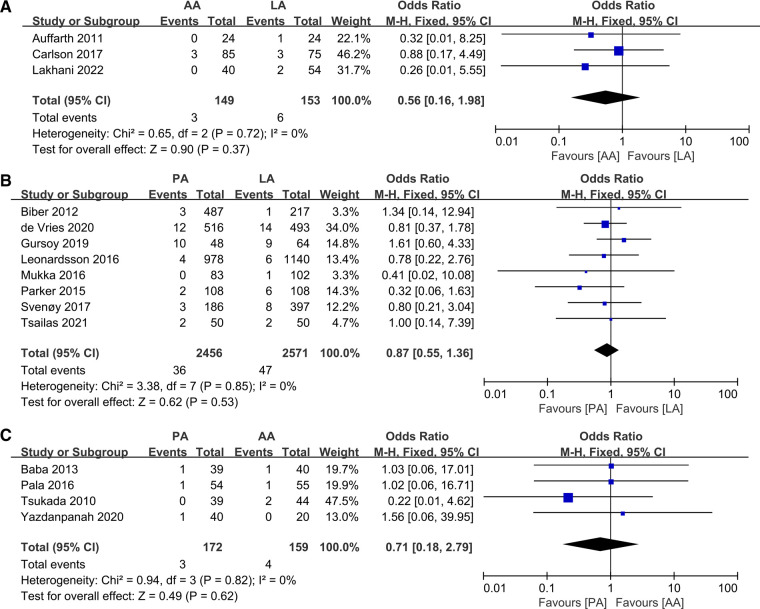
Forest plot comparison of fractures. (**A**) Anterior approach and lateral approach, (**B**) Posterior approach and lateral approach, (**C**) Posterior approach and anterior approach.

A series of 15 studies investigating surgery-related fracture complications were reported. Pooling data from 3 studies (20, 26, 32) comparing LA and AA did not reveal statistically significant differences (OR: 0.56 95% CI: 0.16–1.98 *I*^2^ = 0% *P *= 0.37 Figure 8A), while 8 studies (19, 23, 27, 29, 34, 36, 42, 43) analyzed PA and LA (OR: 0.87 95% CI: 0.55–1.36 *I*^2^ = 0% *P *= 0.53 Figure 8B), and another 4 (22, 39, 44, 45) compared LA with AA (OR: 0.71 95% CI: 0.18–2.79 *I*^2^ = 0% *P *= 0.62 Figure 8C).

### Reoperations

**Figure 9:**
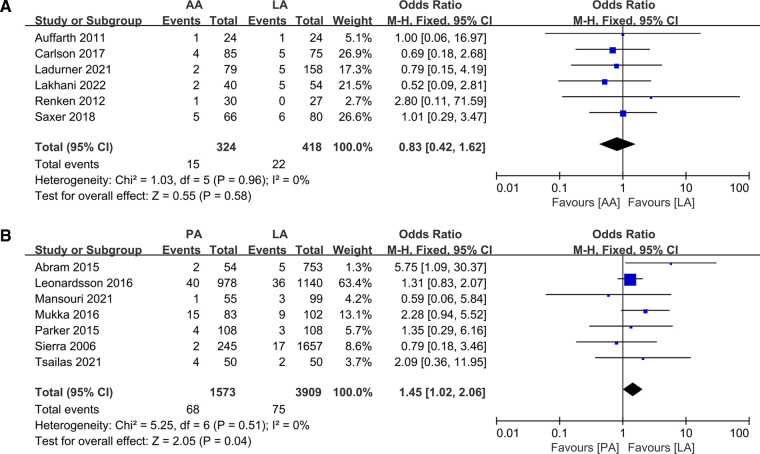
Forest plot comparison of reoperation. (**A**) Anterior approach and lateral approach, (**B**) Posterior approach and lateral approach.

Six papers (5, 10, 18, 20, 26, 32) reported reoperation rates for AA and LA, with no significant heterogeneity between them, and meta-analysis was conducted using a fixed-effects model. The data revealed no statistically significant differences (OR: 0.83 95% CI: 0.42–1.62 *I*^2^ = 0% *P *= 0.58 Figure 9A). Nevertheless, 8 articles (19, 21, 31, 34–36, 41, 43) offered data on PA and LA reoperation, of which Kristensen (31) et al. provided only the rate of postoperative dislocation, without clarifying whether surgical treatment or conservative management was taken after dislocation, and they were excluded from the analysis. The results showed a significant discrepancy (OR: 1.45 95% CI: 1.02–2.06 *I*^2^ = 0% *P *= 0.04 Figure 9B), which may be impacted by the rate of surgical dislocation. Only Langlois (33) et al. compared reoperation rates for PA vs. AA, but no significant difference was reported.

### Mortality

**Figure 10:**
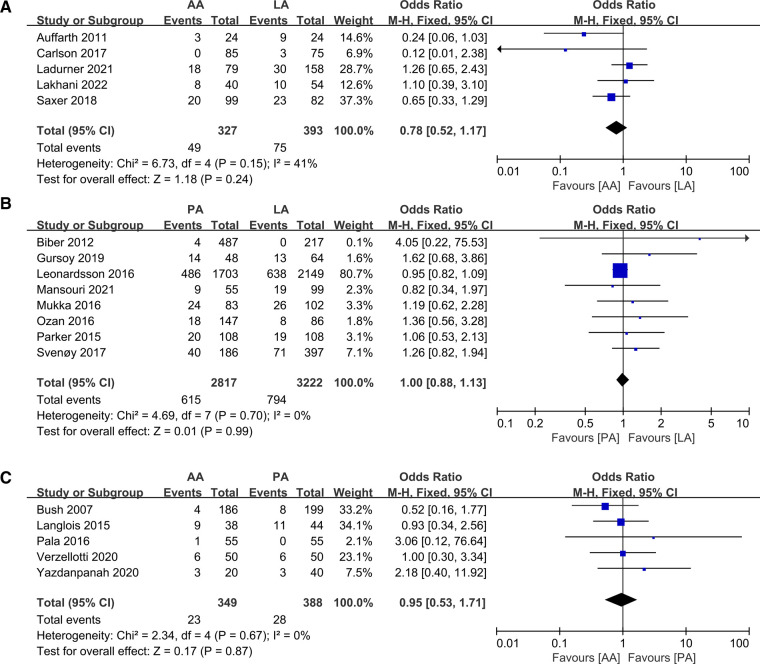
Forest plot comparison of mortality. (**A**) Anterior approach and lateral approach, (**B**) Posterior approach and lateral approach, (**C**) Posterior approach and anterior approach.

Mortality was examined in 17 studies with a total of 7,496 patients and 1,584 deaths. Numerous studies had variable follow-up times for mortality, including 1 month, 3 months, 6 months, 12 months and final mortality, and we adopted the use of most data with 1 year of follow-up for the analysis. There was no significant difference between the groups. The details are as follows, (OR: 0.78 95% CI: 0.52–1.17 *I*^2^ = 41% *P *= 0.24 Figure 10A), (OR: 0.95 95% CI: 0.53–1.71 *I*^2^ = 0% *P *= 0.87 Figure 10B), (OR: 1.00 95% CI: 0.88–1.13 *I*^2^ = 0% *P *= 0.99 Figure 1C), respectively.

## Clinical outcomes

For perioperative pain results, researchers have applied several kinds of scores, including the VAS score (5, 18, 20, 31, 34, 37), a modified Charnley pain score (19), pain numeric rating scale (PNRS) (36), and mean pain postop (NRS) (39) but none of the data could be analyzed in aggregate. In both the AA and PA studies, Pala and Neyisci concluded that the AA group was less painful and that the difference was statistically significant, but Langlois concluded that there was no difference between the groups. Renken (18) et al. and Saxer (5) et al. found that patients in the AA group suffered less pain at all time points than those in the LA group, which may be related to less surgical trauma in the AA group. The difference was that Renken derived a significant difference in pain late (16 days, 40 days), while Saxer considered it to be early (5 days). More importantly, Auffarth (20) arrived at the opposite idea from the two formers.

Hip function was evaluated with the Harris Hip Score (HHS) (20, 36, 45) and the Western Ontario and McMaster Universities Arthritis (WOMAC) (36) index, the University of California, Los Angeles (UCLA) (38) hip scoring assessment, but the number of available databases is insufficient and a meta-analysis of these results is also not feasible. In conclusion, no statistically significant differences were found in all studies, regardless of the functional scores. Lakhani (32) and Sayed-Noor (40) reported the problem of Trendelenburg gait after surgery, Lakhani found no cases of Trendelenburg gait in the DAA group after surgery, while three cases of Trendelenburg gait in the DLA group. Sayed-Noor et al. saw that Trendelenburg sign occurred in 37.5% (9/24) of patients after DL approach and 4% (1/24) of patients after PL approach, further analyzing the DL approach as a factor contributing to Trendelenburg gait.

## Discussion

The different approaches of hemiarthroplasty for femoral neck fractures are used routinely in clinical situations, but their risks and benefits have been controversial. This review is aimed at providing objective theoretical evidence for clinical diagnosis and treatment through meta-analysis of their data. The outcomes revealed that the time of hospitalization was shorter in AA than in LA, and regarding complications, PA had a significantly higher subluxation hazard in comparison to LA and AA, and PA increased the incidence of reoperation than AA, all of which were statistically significant, with no significant differences seen in the remaining outcomes in all aspects that allowed for data analysis.

Heavy heterogeneity existed in clinical data regarding operative time, intraoperative blood loss, and length of hospital stay (*I*^2 ^> 75%), and we used a random-effects model analysis and discovered that AA had a longer length of stay than LA, which may be relevant to the surgical approach.

The AA operation is mostly carried out using the minimally invasive direct anterior approach (DAA), which reduces postoperative pain and allows for faster recovery by preserving the muscles during the approach to the hip joint (46–48), with minimal postoperative impact on hip mobility and daily life (39). Nevertheless, many reports have found a prolonged duration of surgery, although we did not analyze a significant difference (20, 32).

Dislocation was the most disparate outcome in the meta-analysis and an essential indicator for evaluating the results of the surgery. The PA is at greater risk of dislocation than the other two approaches and may be related to surgical incision of soft tissue anatomy such as the capsule, short external rotators, and piriformis (49).There may also be a relationship with patient cognitive status and sex, with some findings of higher dislocation rates in women and patients with cognitive impairment (29).

Patient reoperation was also statistically significant, and one of the major factors was dislocation of the hip on the patient's side of surgery. Some of the surgeries involved repair of the short external rotators with the posterior capsule to reduce the incidence of dislocation, but the efficacy is conflicting (29, 50). This is a shortcoming of PA and may lead the operator to prefer other modalities when choosing a surgical approach.

We did not undertake a meaningful statistical analysis to demonstrate which approach had less postoperative pain (5, 18, 37, 39), but the literature screened generally concluded that AA had better outcomes than PA and LA, which was related to surgical trauma. However, differently, Renken (18) et al. yielded a significant difference in pain between groups at the late stages (16 days, 40 days), whereas Saxer (5) found that the difference occurred at the early stage (5 days).

We can see that PA has more risk of dislocation and reoperation rate, which may be the primary factor for clinicians to avoid. Less of the length of hospitalization is beneficial for AA to be promoted in clinical practice, in addition, whether it can reduce postoperative pain, need to be validated and supported by more clinical trial data in the future.

## Limitations

Although there are more data analyzed in this paper, the randomized controlled trials included in it are only 5, the rest are cohort studies, the quality of evidence is not high level. The double-blind control of these surgical randomization groups and investigators, patients, is comparatively weak, and the likelihood of breaching the blind is higher. Second, different types and manufacturers of prostheses were used for the surgery, and according to the patients' demand, cemented and noncemented prostheses were also employed, all of which had an impact on the experimental results, whereas we were unable to perform a statistical analysis. Third, we only searched common databases and failed to provide comprehensive coverage of published articles, and there were subjective influences of researchers in the selection process of articles, which may lead to the omission of any literature. Furthermore, the included literature reported data for the three approaches, and the number of some indicators was too limited for analysis. For common postoperative hip scores and pain, various scoring criteria used in different studies failed to be analyzed effectively, which is pivotal to the evaluation of the trial, and more literature reporting and unification of routine scoring scales would be more helpful for the study in the future.

## Conclusion

Generally, the risk of dislocation was higher in PA than in the other two approaches, the odds of complications requiring reoperation were also higher in PA than in LA, and PA was not found to be superior to the other two approaches in other aspects. In terms of hospitalization time, AA was shorter than LA, and there was no difference between both in operation time, intraoperative blood loss, all sorts of complications, postoperative pain, and hip function. At present, AA is seemingly more successful, but its literature is limited and has higher heterogeneity. More qualitative literature and postoperative data reported in the future will facilitate the final determination of which approach is superior. The ultimate choice of the operation mode should be considered by the surgical operators based on a combination of factors.
